# SERE: Single-parameter quality control and sample comparison for RNA-Seq

**DOI:** 10.1186/1471-2164-13-524

**Published:** 2012-10-03

**Authors:** Stefan K Schulze, Rahul Kanwar, Meike Gölzenleuchter, Terry M Therneau, Andreas S Beutler

**Affiliations:** 1 Departments of Oncology and Biostatistics, Mayo Clinic, Rochester, MN 55905, USA

**Keywords:** SERE, Simple Error Ratio Estimate, RNA-Seq, Pearson’s correlation coefficient, Replicates, Kappa, Poisson variation, Count data

## Abstract

**Background:**

Assessing the reliability of experimental replicates (or global alterations corresponding to different experimental conditions) is a critical step in analyzing RNA-Seq data. Pearson’s correlation coefficient *r* has been widely used in the RNA-Seq field even though its statistical characteristics may be poorly suited to the task.

**Results:**

Here we present a single-parameter test procedure for count data, the Simple Error Ratio Estimate (SERE), that can determine whether two RNA-Seq libraries are faithful replicates or globally different. Benchmarking shows that the interpretation of SERE is unambiguous regardless of the total read count or the range of expression differences among bins (exons or genes), a score of 1 indicating faithful replication (i.e., samples are affected only by Poisson variation of individual counts), a score of 0 indicating data duplication, and scores >1 corresponding to true global differences between RNA-Seq libraries. On the contrary the interpretation of Pearson’s *r* is generally ambiguous and highly dependent on sequencing depth and the range of expression levels inherent to the sample (difference between lowest and highest bin count). Cohen’s simple Kappa results are also ambiguous and are highly dependent on the choice of bins. For quantifying global sample differences SERE performs similarly to a measure based on the negative binomial distribution yet is simpler to compute.

**Conclusions:**

SERE can therefore serve as a straightforward and reliable statistical procedure for the global assessment of pairs or large groups of RNA-Seq datasets by a single statistical parameter.

## Background

Massively parallel shotgun RNA-Sequencing (RNA-Seq) has become the technology of choice for transcriptome analysis because of its potential to yield extensive biological information with digital precision. The development of effective statistical data analysis methods has been essential to the utility of RNA-Seq and has been a focus since the original reports on the technology [1,2]. The statistical analysis of RNA-Seq variability has been the focus of several comprehensive studies [3,4] and remains a topic of active investigation [5]. A common task in RNA-Seq statistical analysis is to determine whether two RNA-Seq datasets are faithful replicates and, if not, whether two datasets differ only slightly or very markedly. Sophisticated statisitical tools for analysis of Next-Generation Sequencing data are beginning to appear, e.g. the edgeR package [6] and DESeq package [4]. Here we focus on a more targeted approach that is useful in both quality control and early analysis. An ideal measure for this task should be easy to compute and have three features: (1) Sensitivity: The measure is sensitive to actual differences; (2) Calibration: There is a known baseline value that corresponds to success; (3) Stability: The behavior is independent of the sequencing depth, total number of exons, and other experimental conditions not relevant to the question. Simple computation is a desirable but not essential attribute.

Pearson’s correlation coefficient *r* has been widely used to affirm that pairs of RNA-Seq datasets are faithful replicates [1,2,7-9] and continues to be in use [10-12]. However, as a quasi standard in the RNA-Seq literature *r* may be problematic as it may suffer not only from the general pitfalls that have long been recognized (e.g. Chambers et al. [13]) but from additional shortcomings specific to count data.

As an alternative, McIntyre et al. recently suggested a measure of concordance based on the Kappa statistic to compare RNA-Seq samples [5]. Applying the Kappa procedure to multiple RNA-Seq samples, the authors concluded that replicates of the same RNA-Seq library from different “lanes” (compartments in an Illumina genome analyzer flow cell used to separate samples) were subject to a systematic bias, a finding that appeared to contradict previous observations by others [3,4,14]. Similarly to Pearson’s *r*, Kappa may be subject to confounding factors of the experimental design such as the total read count. Furthermore, interpreting Kappa under the premise that it should be 1 for perfect replicates may be naive.

Here we propose a new candidate statistic for RNA-Seq sample comparison based on the ratio of observed variation to what would be expected from an ideal Poisson experiment. We show that the Simple Error Ratio Estimate (SERE), unlike *r* and Kappa can be expected to be 1 for perfect replicates, only affected by the random sampling effect. We evaluated the 3 statistics on the above criteria (calibration, sensitivity, stability) using original RNA-Seq data from rat neural tissue that contained multiple technical and biological replicates. From this we created ideal in silico replicates by randomly splitting an observed lane into 2 pseudo-lanes and also simulated various degrees of contamination. This allowed us to examine the behavior of the methods under known outcomes and revealed serious deficiencies in the correlation and concordance approach. Finally the methods were compared on the actual datasets.

## Results and discussion

### Candidate statistical measures

Pearson’s correlation coefficient *r* was the main competitor because of the ubiquity of its use in the current RNA-Seq literature. The Kappa statistic was recently proposed as an alternative [5]. This requires the counts to be binned. Here, we used the same binnig as suggested in that paper. The SERE statistic is a ratio of the observed standard deviation between replicates divided by the value that would be expected from an ideal experiment.

### Sensitivity experiment

**Figure 1:**
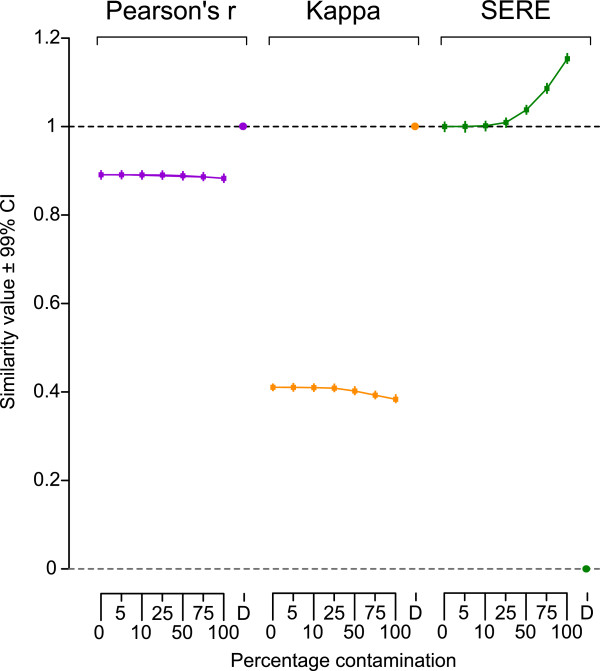
**Sensitivity and Calibration analysis of candidate statistics on simulated contamination and duplicated replicates RNA-Seq datasets.** One in silico replicate out of a pair was successively contaminated by reads from a biological replicate. Pearson’s *r* and Kappa showed no obvious changes between different degrees of contamination and perfect replication (0% contamination). SERE on the contrary was sensitive as early as 25% of the reads were originated from the biological replicate. Marked differences appeared as soon as the contamination reached 50% (SERE = 1.04). For duplicated datasets (“D”, identical data), which can either result from a “copy and paste” error or data falsification, Pearson’s *r* and Kappa are 1.0 suggesting perfect replication, although duplicates are imperfect replicates. SERE clearly discriminates between duplicated replicates (SERE = 0.0) and perfect replicates (SERE = 1.0). All computations were performed on RNA-Seq sample “control 1”, which was randomly split in a pair of in silico replicates (5 million reads per sample). Then, one in silico sample was contaminated to different degrees by reads originating from “control 2”. The procedure was repeated 200 times. D: duplicates.

Figure 1 shows results from constructed datasets representing two lanes from an ideal replication experiment (“perfect” in silico replicates affected only by the random sampling effect), and pairs of lanes where one of the two has various amounts of contamination. The contaminating sample in this experiment was from a litter mate under the same experimental condition (biological replicate) in order to make detection purposefully difficult. Each point of the figure represents the average of 200 independent realizations of the simulation experiment. The last point for each method represents a duplication, i.e., comparing a lane with itself (This could be the result of a copy/paste error during analysis, for instance).

For the correlation and concordance measures the value 1 is usually viewed as the “ideal”. This is only achieved for the duplication, a situation where the randomness inherent to the process of read sampling is not allowed and instead a greater than expected congruence between two sample pairs is forced, resulting in an extreme case of “underdispersion”. Data from an actual ideal experiment (0% contamination) had on average correlation values of 0.89 and concordance values of 0.41. SERE on the contrary yielded the expected baseline value of 1 for perfect in silico replicates (0% contamination) and detected contamination as early as 25%. Marked differences appeared when contamination reached 50%. The SERE measure also clearly marks the duplication comparison as unusual (SERE = 0). The sensitivity of both the correlation and concordance measures is much lower, making it difficult to distinguish contaminated samples from the ideal experiment.

### Stability experiment

**Figure 2:**
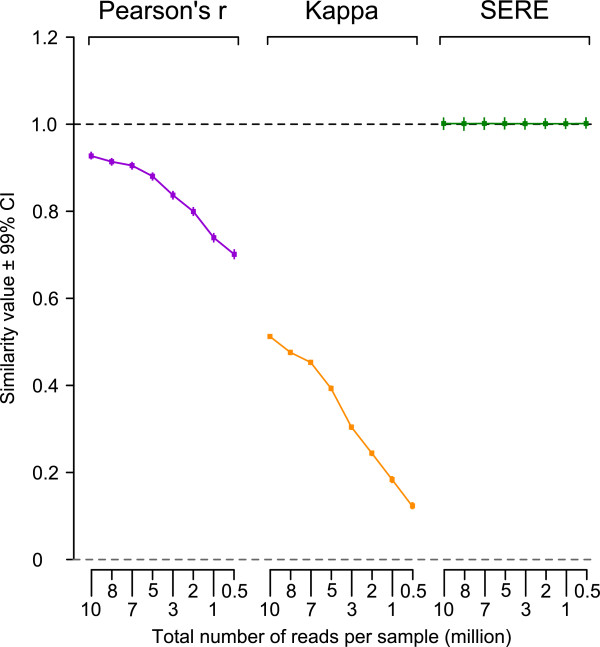
**Total read count (sample size) dependence of candidate statistics comparing perfect replicate RNA-Seq datasets.** The Simple Error Ratio Estimate (SERE) was 1 when two replicate RNA-Seq datasets of different sizes were compared. Variation of SERE for repeat computations from independent replicate dataset pairs for each total read count demonstrated a stable 99% confidence interval (CI) of approximately +/- 0.01. The Pearson correlation coefficient fell as read counts decreased. Kappa also strongly depended on the total read count. All computations were performed on 200 model RNA-Seq datasets obtained by drawing reads randomly from a universal read set (described in Methods).

Another characteristic, stability, interrogates whether the behavior of the underlying statistic is independent of ancillary aspects of the experiment; the obvious such factor in RNA-Seq is the sequencing depth. Therefore, RNA-Seq perfect replicate datasets of different sizes were generated by drawing random reads from the universal read pool. We simulated two types of scenarios: In our first experiment (Figure 2) we decreased the number of reads in both lanes from 10 to 0.5 million. Pearson’s *r* fell markedly from 0.93 to 0.71 when the read counts of both datasets in a pair were reduced (Figure 2). Kappa was equally sensitive to the total read count, decreasing from 0.54 for perfect replicate pairs with 10^7^ reads per sample down to 0.09 for pairs with only 0.5x10^6^ reads. All datasets represented perfect replicates by definition as they were generated in silico by sampling from a common pool. Therefore, low values of Pearson’s *r* such as <0.8 and Kappa <0.3 are not in all cases indicative of poor RNA-Seq experimental replication. SERE was unaffected by the total count of RNA-Seq reads, remaining stable at 1.0.

**Figure 3:**
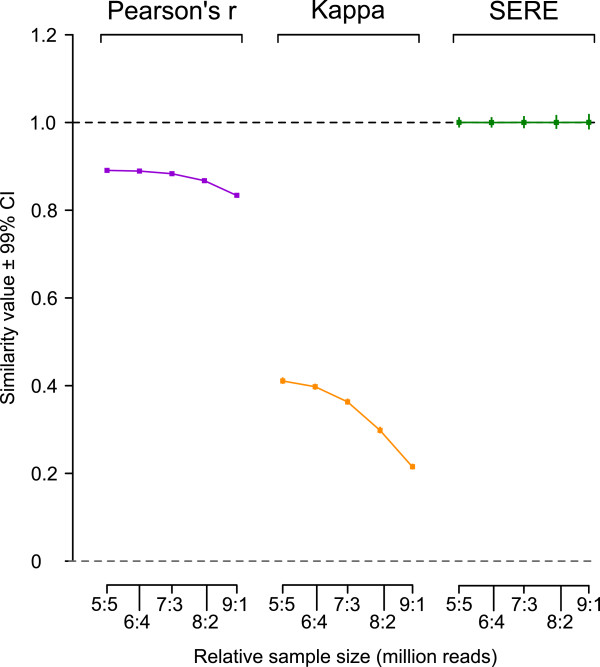
**Impact of unequal sample sizes.** Pairwise comparisons of perfect in silico replicate RNA-Seq datasets were made similarly to Figure 2, but keeping the total read count for each pair constant while systematically varying the relative size of both datasets, e.g., 1x10^6^ plus 9x10^6^, 3 x10^6^ plus 7 x10^6^, 5 x10^6^ plus 5 x10^6^. Pearson’s *r* and Kappa fell for unequal sample sizes demonstrating a maximum for equal sample sizes. SERE remained stable at 1.0 while the 99% CI of repeat measures was optimal (smallest) for equal sample sizes.

In our 2nd experiment (Figure 3) we kept the total read count of 200 in silico replicates constant, but varied the relative size of both samples, simulating multiplexed RNA-samples, where both samples will not always yield the same number of reads. Pearson’s *r* and the Kappa statistic performed continuously worse, as the relative difference between the two perfect replicates became bigger, reaching values of approximately 0.82 and 0.19 respectively at the extremes (1 million versus 9 million uniquely mapped reads per sample). SERE on the contrary stayed at a stable value of 1.0 through all the scenarios. A minor increase of the confidence intervals could be observed as the relative sample size tended to the extremes, yet remaining between ± 0.01.

### Performance of the statistics on empirical data

**Figure 4:**
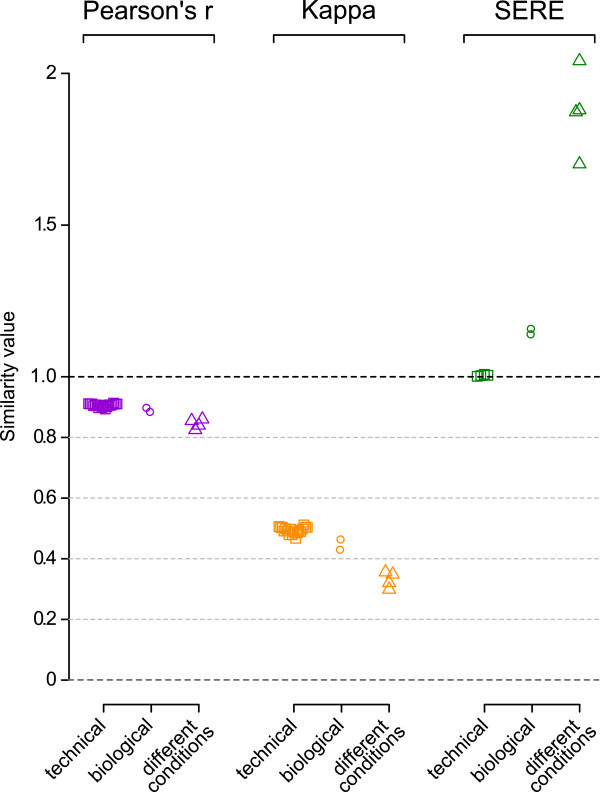
**Benchmarking of the test statistics on empirical RNA-Seq data.** Three scenarios were investigated: technical replicates (different lanes from the same RNA-Seq library); biological replicates (different RNA-Seq libraries but same experimental condition); experimental differences (RNA-Seq libraries from different experimental conditions). Pearson’s *r* and Kappa diverged for the three scenarios but differences were small compared to the impact of varying total read numbers (see Figures 2 and 3). SERE allowed for a straightforward categorization of the three scenarios: technical replicates representing normal/near-normal dispersion were 1; biological replicates representing moderate overdispersion and different experimental conditions representing marked overdispersion ranged from 1.14 to 1.16 and 1.7 to 2.0 respectively, i.e. >>1. For *r* and Kappa lanes were compared pairwise, whereas for SERE multiple lanes were compared simultaneously. For the biological replicates comparisons and the different experimental conditions, we selected the first lane of each sample as an example to evaluate *r* and Kappa. For SERE all lanes were included.

To put the above findings into perspective, we studied the candidate statistics on an empirical dataset which included technical and biological replicates, as well as samples from different experimental conditions (“control” vs. “SNL”, see Methods). Figure 4 shows the result for 14 lanes of data, consisting of 3 replicate lanes for each of the 2 “control” rats and 4 replicate lanes for each of the 2 “SNL” rats. First the replicate lanes of each rat were compared (technical replicates), second the biological replicates (the 2 “controls” and the 2 “SNL” respectively) and third the animals belonging to different experimental groups (“SNL” vs. “control” rats). Note that SERE results in a single value for a set of lanes that are being compared, while the correlation and concordance measures apply only to pairs of lanes.

*R* and Kappa were slightly lower for the biological replicates as compared to the technical replicates and further decreased when comparing the two experimental conditions. However, differences were small compared with those caused by total read counts (Figures 2 and 3) suggesting that both measures are poor candidates for detection of global alterations in practice. SERE was highly sensitive to global differences, with scores of approximately 1.15 for biological replicates and 1.7 to 2.0 for comparisons between different experimental conditions.

**Figure 5:**
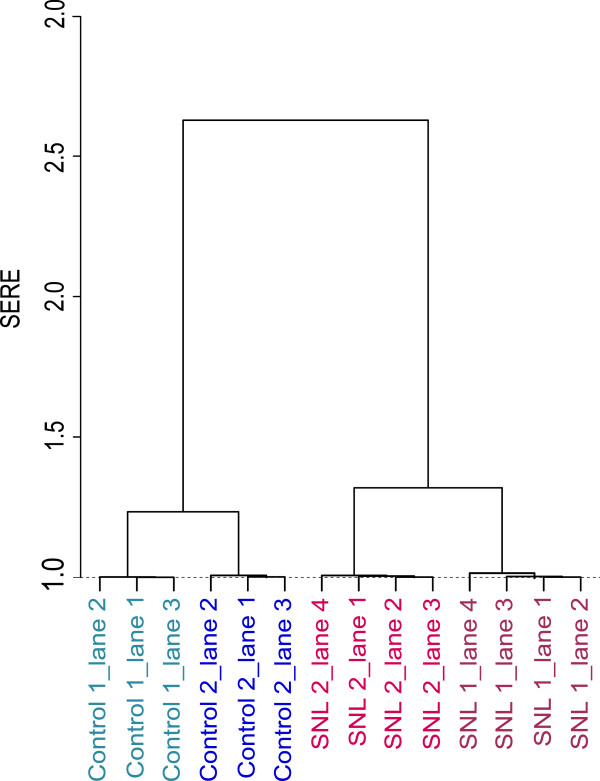
**SERE as a measure for clustering.** RNA-Seq datasets could be meaningfully clustered using SERE, indicating that it is a practical and useful test statistic if the similarity or global differences between many samples of RNA-Seq datasets need to be characterized by a single global paramater.

The SERE statistic can also be computed pairwise. For the 3 technical replicates of “control 1” for instance, the overall ratio for the three lanes is 1.005, with pairwise values of 1.003, 1.002, and 1.008. When the overall SERE statistic for a set of lanes is large we can use these individual comparisons to further sort out which lane(s) is the source of concern. A simple way to display this is to use SERE to create a cluster map. Figure 5 shows the resulting dendrogram for the 14 lanes of data used in Figure 4. The dendrogram clearly reflects to the experimental design by first distinguishing between the two conditions, then separating the biological replicates within an experimental configuration and finally grouping the technical replicates. The vertical axis of dendrograms is often left unlabeled since the values are on an arbitrary scale, but in this case they have a direct interpretation as “excess dispersion”. A more interesting example is shown in Additional file 1: Figure S1 where we applied SERE on a drosophila melanogaster dataset (SRA id GSE17107) that was employed by McIntyre et al. Interestingly, it revealed 2 distinct groups (no sign of overdispersion within the groups) although the 5 samples originated from the same RNA-Seq library suggesting a possible batch effect.

### The drawbacks of *r* and Kappa

This study was focused on a global approach that is useful in both quality control and early analysis of RNA-Seq experiments. Therefore, an ideal measure for this task was defined to be easy to compute and have three features of sensitivity, calibration and stability. The SERE measure does well, but the correlation and concordance have serious flaws. Why?

Deficiencies in the correlation coefficient have long been known. Chambers et al. [13] for instance showed a panel of 8 graphs with very different patterns all with the same value of *r*. Of most relevance here is that *r* can be dominated by values at the extremes of the data. Count data for RNA-Seq is very skewed. For example, in the first lane of “control 1”, 55.4% of the observed exons had a count of 10 or less out of 5,512,030 million total uniquely mapped reads, while the highest had a count of 95660. Even under a log-transformation, the largest few counts have in inordinate influence. Further examination of the results underlying Figures 2 and 3 shows that the value of *r* for the ideal samples is essentially determined by the range of the counts, which in turn is closely related to the total sequencing depth (Additional file 1: Figure S2). This causes *r* to have both a varying target value (the expected result for a perfect replicate) and high variability. Even a low value (e.g <0.8) might result from an “ideal” experiment. At the same time, markedly different samples can yield correlation coefficients of >>0.8 if the total read count is high. Moreover, Pearson’s *r* can be computed in several ways: on square root of the raw counts, on count data after addition of a pseudo-count and log-transformation or on RPKM data after adding a pseudo-count and log-transformation. The latter is the most commonly used normalization and therefore employed here. These different approaches can substantially change the outcome since they influence the distance between the extremes (data not shown).

By categorizing the data into bins, as performed by the Kappa statistic, one avoids the susceptibility to values on the extreme of the scale. However the choice of the bin sizes becomes the driving factor for this statistic. Additional file 1: Figure S3 demonstrates how the binning influences the result of Kappa for one and the same dataset. If the bins are chosen as reported previously by McIntyre et al. (Additional file 1: Figure S3A) Kappa is 0.41. When the bins are chosen wider (Additional file 1: Figure S3D), the value for Kappa raises to approximately 0.73. By choosing very small bin sizes (Additional file 1: Figure S3C), the Kappa value decreases to approximately 0.3. We also computed a weighted Kappa [15] employing the most common definition where disagreement is proportional to the distance from the diagonal. The result shown in Additional file 1: Figure S4 demonstrated the same characteristics of Kappa shown for the unweighted procedure.

For the simulation study, we chose the unweighted Kappa. We took the same bin sizes as proposed by McIntyre et al., which used 0 counts as the smallest bin. Therefore, whenever the expression of an exon is so sparse that only a single read is detected among two or more samples (the exon is a singleton) the exon will be scored as “off the diagonal” since it will fall into the bin “0” for one sample and in “1-10” for the other sample. The fraction of singletons in our in silico samples with 5 million UMRs is 11.56-11.96%, which alone limits the Kappa to a maximum of about 0.89. The total fraction of singletons tended to decrease by increasing the total read count and the calculated Kappa value rises as seen in Figure 2.

Computational simulation can be helpful in estimating the expected values for Pearson’s *r* or Kappa but need to take the specific experimental condition into account and cannot be generalized easily. Therefore the use of *r* and Kappa to investigate whether two RNA-Seq datasets are faithful replicates or subject to systematic differences or bias leads to ambiguity in most cases.

### The Simple Error Ratio Estimate (SERE)

The third candidate statistic appears to be a useful measurement to identify global differences between RNA-Seq data by fulfilling the set criteria of a good measure. A primary reason is that it compares the observed variation to an expected value, and the latter accounts for the impact of varying read depth. It is easy to compute and satisfies our three primary criteria.

#### Calibration

A “perfect” SERE of 1 indicates that samples differ exactly as would be expected due to Poisson variation. If RNA-Seq samples are truly different, this is identified by values > 1 (overdispersion). Values below 1 are well interpretable and indicate “underdispersion,” e.g. through artefactual duplication of data. A value of 0 would constitute perfect identity, such as might occur from accidentally duplicating a file name. Interestingly, detection of underdispersion has been important in detecting data falsification [16]; faithful randomness is difficult to fabricate.

#### Sensitivity

A constructed replicate with 25% contamination was successfully indicated as overdispersed by SERE. As soon as one dataset contains 50% of its reads from another biological replicate, the indication of overdispersion becomes even more obvious. Thus, SERE is a qualified measure to detect processing errors and other sources of variation.

#### Stability

In RNA-Seq experiments the read counts per exon in a sample vary, either due to rareness of the exon within the sample or due to total number of reads. The expected variation between lanes for that exon also changes. Because SERE explicitly accounts for this, comparing observed to expected counts, it is largely unaffected by these changes, regardless of the sequencing depth. This was confirmed by 200 in silico simulations performed for various numbers of reads, where SERE was 1 on average. However, each simulation is subject to variation and therefore will slightly deviate from 1 either in the direction of under- (<1) or overdispersion (>1). To characterize the range of this variation we calculated the confidence interval (CI) for all the simulations. As seen in Figure 2, the 99% CI was narrow, ranging from 0.99 to 1.01 regardless of the total read count.

As shown in Figure 5, we can also use the measure to more finely dissect the variation found in an experiment. This is a useful extension to the quality control assessment. However, when comparing samples on a single gene level, e.g. multiple treatment groups, both the expected Poisson variation exploited by SERE and the biological variation between and within treatment groups play an important role, and methods that take both into account would be preferred for deeper inquiry (e.g. DESeq package of Anders et al. and edgeR by Robinson et al.). Yet, SERE remains useful as an initial diagnostic tool.

Li et al. recently introduced the “Irreproducible Discovery Rate” (IDR) as a measure of reproducibility [17] and demonstrated its utility for the analysis of a variety of sequencing-based high-throughput data types [18]. We tested the IDR on two scenarios considered in this study, namely a comparison of identical datasets and a comparison of perfect replicates. In the case of identical datasets, the change of correspondence curve generated by IDR analysis indicated perfect correspondence (Additional file 1: Figure S5) lacking an indicator that this is a case of extreme underdispersion indicative of an unexpected finding such as human error or mischief. In the case of perfect replicates (generated in silico and differing only due to Poisson variation) the change of correspondence curve became noticeably noisy when the total number of read counts in the simulation was small (Additional file 1: Figure S6). The IDR was developed for comparison of datasets with unknown or differing distribution types. It is therefore unsurprising that it appears to be less useful than SERE for the detection of under- and overdispersion in the comparison of replicate datasets affected by Poisson variation. IDR was developed for situations where two experimental replicates or methods could reasonably expected to agree without anticipating perfection. The statistical concept underlying SERE is unrelated to IDR and was developed for situations where two experimental replicates could reasonably be expected to differ only due to stochasticity.

## Conclusions

SERE provides an efficient single-parameter statistical measure of reproducibility for RNA-Seq datasets. Unlike two other measure currently in use, Pearson’s correlation coefficient *r* and the concordance measure based on Kappa statistic, SERE is independent of typically varying experimental circumstances (such as the total count of reads). The interpretation of SERE is straightforward staying clear of the ambiguities resulting from misinterpretation of *r* and Kappa. A SERE of 1.0 corresponds to the normal degree of dispersion resulting from the Poisson variation of raw read counts. Because SERE is a measure of dispersion, its interpretation extends to two situations of practical importance: Underdispersion indicative of for example data duplication and overdispersion usable as a global measure of the degree of alterations, which is agnostic to the relative importance of difference genomic regions because SERE weights each observed read identically. SERE may in principle be applicable to a broad range of read count datasets such as from CHIP-seq or for comparison of alternately processed read data such as the counts of a k-mer analysis. The present study suggests that SERE has superior characteristics to previously used measures in the case of RNA-Seq.

## Methods

### Empirical RNA-Seq data

RNA-Seq read data used in the present analysis was taken from a previous study [8] and is available through the sequence read archive (SRA id GSE20895). 14 RNA-Seq datasets were used, each corresponding to the sequencing reads from one “lane,” which is a physical compartment in the flow cell of the Illumina GA-II instrument. The 14 lanes corresponded to 4 RNA-Seq libraries, whereby 3 lanes of sequence data were available for each of two of the libraries and 4 lanes for each of the other two. Each library was synthesized from a different source of RNA. RNA sources corresponded to two experimental conditions, “SNL” (spinal nerve ligation) or “control.” Two independent RNA-Seq libraries, “biological replicates,” were available for each condition. The published analysis [8] found that biological replicates were similar and that the 2 experimental conditions were meaningfully different. Technical replicates were not compared in the previous report (instead reads originating from different lanes corresponding to the same RNA-Seq library were pooled) but are included in the comparisons made in the present study.

Additional file 1: Table S1 lists the condition and replicate identification corresponding to each of the 14 lanes. The table illustrates how the available data allowed for three types of comparisons between RNA-Seq datasets: pairs of technical replicates (same library sequenced on different lanes); pairs of biological replicates (same experimental condition); and the two experimental conditions “SNL” *versus* “control”.

### Mapping and annotation

RNA-Seq reads (50bp) were aligned to the rat reference genome (RGSC 3.4) by Bowtie [19]. We allowed for a maximum of two mismatches and considered only the uniquely mapped reads for the downstream analysis. This filtration step resulted in 108,636,496 million UMRs to the genome. Genome annotation ENSEMBL 65 was used including 22,921 protein coding genes. Overlapping exons of genes having multiple isoforms were combined resulting in a total of 222,097 exons (Additional file 2). For the subsequent analysis exons served as bins, i.e., reads aligning to each exon were counted and the sum noted in the master read count file (Additional file 3). The file has 222,097 rows (bins corresponding to exons) and 14 columns (corresponding to each of the lanes described above).

### A universal pool of RNA-Seq reads for the simulation experiments

All uniquely mapped reads (from lane 1 to 3) from the first “control” RNA-Seq sample were combined resulting in 22.9 × 10^6^ reads in order to create a universal pool. The datasets for the in silico duplicates and replicates described below were generated from this pool. The in silico replicates created from the universal pool of RNA-Seq reads by random drawing are by definition only different due to stochastic (Poisson) variation of the sampling process (see Results). Similarly all 3 lanes from “control 2” were combined to create a second pool used as “contaminant” in the contamination experiments.

### In silico replicates: “Perfect” replicates

A set of RNA-Seq datasets faithfully representing Poisson variation only (perfect non-identical replicates) was generated by randomly choosing sets of 5 x10^6^ reads from the universal pool by using the “sample” function in R (Additional file 4) and a Java script (Additional file 5). This process was repeated 200 times and used as reference of perfect replicates in the subsequent benchmarking of the different test statistics. The same procedure was further repeated with different set sizes (0.5 to 10 million reads per sample) in order to test for the influence of total read counts on the statistics.

### In silico contamination

To test whether the statistical measures were sensitive to actual differences, we contaminated one in silico replicate out of a pair with 0;5;10;25;50;75;100% of a biological replicate (“control 2”) via computer simulation. In detail, the first sample was created by randomly drawing 5 million reads of the universal pool of “control 1” and the second by drawing x% of reads from “control 1” and y% of reads from file “control 2”, whereby x+y=100, corresponding to 5 million reads. The procedure was repeated 200x.

### Processing of the empirical data

For Pearson’s correlation coefficient and Kappa, the 3 lanes of each of the two “control” and the 4 lanes for each of the two “SNL” condition were compared in a pairwise fashion, resulting in a total of 18 technical replicate comparisons (see Figure 4). To compare the performance of the statistics on biological replicates we compared the first lane of “control 1” to the first lane of “control 2”, and lane 1 from “SNL 1” to lane 1 from “SNL 2”. To test whether Pearson’s *r* and Kappa were sensitive to different experimental conditions, we selected the first lane of each “control” and compared it to the first lane of each “SNL” sample. SERE is not restricted to pairwise comparisons, but allows to compare multiple samples or lanes simultaneously. Therefore, SERE yielded 4 values for the technical replicate comparisons, 2 for biological and 4 for different experimental conditions.

### Simple Error Ratio Estimate (SERE)

Given a set of N exons and M lanes, let *y*_*ij*_ denote the number of reads covering the *i*^*th*^ exon in the *j*^*th*^ lane. Let *L*_*j*_ be the total read count for lane *j*, *E*_*i*_ the total for exon *i*, and T the grand total count across all lanes and exons. Under the hypothesis that the lanes are simple technical replicates of each other, they will have a Poisson distribution with one parameter. This parameter can be thought of as the expected number of reads for the lane *j* and the exon *i*. Its estimate can be calculated using eq. 1.

$${\hat{y}}_{\mathrm{ij}}=\frac{E_{i}L_{j}}{T}$$

The expected variation for each observation *y*_*ij*_ is ${\left(y_{\mathrm{ij}}-{\hat{y}}_{\mathrm{ij}}\right)}^{2}$, and the expected variation under the Poisson assumption is ${\hat{y}}_{\mathrm{ij}}$. This gives a per exon overdispersion estimate of:

$$s_{i}^{2}=\frac{1}{M-1}\sum_{j}\frac{{\left(y_{\mathrm{ij}}-{\hat{y}}_{\mathrm{ij}}\right)}^{2}}{{\hat{y}}_{\mathrm{ij}}}$$

The denominator is (*M* − 1) due to the constraint that $\sum_{j}\left(y_{\mathrm{ij}}-{\hat{y}}_{\mathrm{ij}}\right)=0$ for each exon *i*.

Averaging over all N exons we have:

(3) $$s^{2}=\frac{1}{N}\sum_{i}s_{i}^{2}$$

The SERE estimate is $s=\sqrt{\left(s^{2}\right)}$.

Simple algebra shows that for a singleton count, i.e., an exon that appears only once in only one of the lanes, SERE equals exactly 1. That is, singletons shrink the overall SERE estimate towards 1, whether or not the samples are actually replicates. Therefore, we modify the average in equation 2 to sum over only the non-singleton counts. The R code to calculate SERE is provided in Additional file 6.

The unmodified measure of equation 2 is the measure of Poisson over-dispersion most often used in generalized linear models, see for instance the classic textbook of McCullagh and Nelder [20]. An alternative measure of overdispersion is based on the deviance statistic. Brown and Zhao [21] consider this measure along with two others in the context of random arrival data, e.g. calls to a support center, and show that it is inferior to *s*_*i*_^2^ (equation 2) whenever there are small values for ${\hat{y}}_{\mathrm{ij}}$. Their work corresponds to the case where all lanes have the same total count *L*_*j*_; extending their method shows that (*M* − 1)*s*_*i*_^2^ will be distributed as a chi-square random variable with (*M* − 1) degrees of freedom and non-centrality parameter $\sum L_{j}^{2}{\left(p_{\mathrm{ij}}-{\bar{p}}_{i}\right)}^{2}$. The parameter *p*_*ij*_ is the true fraction of exon *i* within sample *j* and ${\bar{p}}_{i}$ the fraction of exons in the (hypothetical) pooled sample. Under the null hypothesis of sample equality $p_{\mathrm{ij}}={\bar{p}}_{i}$ the non-centrality parameter is zero. *N*(*M* − 1)*s*^2^ will follow a chi-squared distribution with *N*(*M* − 1) degrees of freedom. This can be used to set 99 % confidence intervals on the non-centrality parameter, and through that on the SERE estimate. We provide the corresponding R code in Additional file 7. Bins containing a read count of 0 in both samples of a pair contain no information and were therefore excluded from the comparative analyses of SERE and the two alternative parameters described below.

### Pearson’s correlation coefficient

For a pair of lanes, we calculated the RPKM [1] for each lane. In the RPKM calculation we added a pseudo count of one read to each of the exons. The RPKM values were then log transformed and the Pearson’s correlation was calculated. The process was repeated using the raw counts to verify any influence of the RPKM transform itself, and using both the log and the square root of the raw counts. The latter is the variance stabilizing transform for Poisson data. None of these had any substantive impact on the results (data not shown).

### Cohen’s simple Kappa statistic

The read counts were normalized to RPKM and divided into 9 bins of size: 0, 1-10, 11-20, 21-40, 41-80, 80-160, 161-320, 321-1000 and greater than 1000 RPKM as it was suggested by McIntyre et al. In order to compare a pair of replicates, a 9 × 9 table of counts was constructed, whereby each exon pair added to a cell of the table (see Additional file 8). In this way, exons that were in agreement added to the diagonal of the table, whereas the total fraction of off-diagonal entries contributed to a measure of non-agreement.

## Abbreviations

RNA-Seq: RNA-Sequencing; SERE: Simple error ratio estimate; *r*: Pearson’s correlation coefficient; SNL: Spinal nerve ligation; UMR: Uniquely mapped reads; bp: Base pair; RPKM: Reads per kilobase of exon model per million mapped reads; CI: Confidence interval; IDR: Irreproducible discovery rate.

## Competing interests

The authors declare that they have no competing interests.

## Authors’ contributions

SKS, RK and MG performed the analyses and prepared the figures. TMT and ASB conceived the research and wrote the manuscript. All authors read and approved the final manuscript.

## Supplementary Material

Additional file 1 Contains the supplementary table and figures. Click here for file

Additional file 2 **Is a table listing the exon boundaries for the *rat *annotation.** Click here for file

Additional file 3 Is a master read count table listing the number of reads for each exon in each of the 14 lanes. Click here for file

Additional file 4 **Is an R script to create a hash index file by the ‘sample’ function in R that serves as input for** Additional file 5. Click here for file

Additional file 5 Is the JAVA script to create the in silico replicates. Click here for file

Additional file 6 Is the R code to calculate SERE. Click here for file

Additional file 7 Is an R script to calculate the confidence intervals for SERE. Click here for file

Additional file 8 Is the R code for the Kappa statistic on RPKM scale. Click here for file

## References

[B1] Mortazavi A Williams BA Mccue K Schaeffer L Wold B Mapping and quantifying mammalian transcriptomes by RNA-Seq Nat Methods 2008 5 7 621 628 10.1038/nmeth.1226 18516045 PMC13303166

[B2] Nagalakshmi U Wang Z Waern K Shou C Raha D Gerstein M Snyder M The transcriptional landscape of the yeast genome defined by RNA sequencing Science (New York, N.Y.) 2008 320 1344 1349 10.1126/science.1158441 PMC2951732 18451266

[B3] Bullard JH Purdom E Hansen KD Dudoit S Evaluation of statistical methods for normalization and differential expression in mRNA-Seq experiments BMC Bioinforma 2010 11 94 10.1186/1471-2105-11-94 PMC2838869 20167110

[B4] Anders S Huber W Differential expression analysis for sequence count data Genome Biol 2010 11 R106 10.1186/gb-2010-11-10-r106 20979621 PMC3218662

[B5] McIntyre LM Lopiano KK Morse AM Amin V Oberg AL Young LJ Nuzhdin SV RNA-seq: technical variability and sampling BMC Genomics 2011 12 293 10.1186/1471-2164-12-293 21645359 PMC3141664

[B6] Robinson MD McCarthy DJ Smyth GK edgeR: a Bioconductor package for differential expression analysis of digital gene expression data Bioinformatics (Oxford, England) 2010 26 139 140 10.1093/bioinformatics/btp616 PMC2796818 19910308

[B7] Cloonan N Forrest ARR Kolle G Gardiner BBA Faulkner GJ Brown MK Taylor DF Steptoe AL Wani S Bethel G Robertson AJ Perkins AC Bruce SJ Lee CC Ranade SS Peckham HE Manning JM Mckernan KJ Grimmond SM Stem cell transcriptome profiling via massive-scale mRNA sequencing Nat Methods 2008 5 613 619 10.1038/nmeth.1223 18516046

[B8] Hammer P Banck MS Amberg R Wang C Petznick G Luo S Khrebtukova I Schroth GP Beyerlein P Beutler AS mRNA-seq with agnostic splice site discovery for nervous system transcriptomics tested in chronic pain Genome Res 2010 20 847 860 10.1101/gr.101204.109 20452967 PMC2877581

[B9] Tang F Barbacioru C Nordman E Li B Xu N Bashkirov VI Lao K Surani MA RNA-Seq analysis to capture the transcriptome landscape of a single cell Nat Protoc 2010 5 516 535 20203668 10.1038/nprot.2009.236 PMC3847604

[B10] Beane J Vick J Schembri F Anderlind C Gower A Campbell J Luo L Zhang XH Xiao J Alekseyev YO Wang S Levy S Massion PP Lenburg M Spira A Characterizing the impact of smoking and lung cancer on the airway transcriptome using RNA-Seq Cancer Prev Res (Phila) 2011 4 803 817 10.1158/1940-6207.CAPR-11-0212 21636547 PMC3694393

[B11] Head SR Komori HK Hart GT Shimashita J Schaffer L Salomon DR Ordoukhanian PT Method for improved Illumina sequencing library preparation using NuGEN Ovation RNA-Seq System Biotechniques 2011 50 177 180 21486238 10.2144/000113613

[B12] Gertz J Varley KE Davis NS Baas BJ Goryshin IY Vaidyanathan R Kuersten S Myers RM Transposase mediated construction of RNA-seq libraries Genome Res 2012 22 134 141 10.1101/gr.127373.111 22128135 PMC3246200

[B13] Chambers JM Cleveland WS Tukey PA Klein B Graphical Methods for Data Analysis 1983 Belmont, CA: Wadsworth International Group

[B14] Marioni JC Mason CE Mane SM Stephens M Gilad Y RNA-seq: An assessment of technical reproducibility and comparison with gene expression arrays Genome Res 2008 18 1509 1517 10.1101/gr.079558.108 18550803 PMC2527709

[B15] Fleiss JL Levin B Paik M Fleiss JL Levin B Paik MC Statistical Methods for Rates and Proportions 2003 3 New Jersey: Wiley, John

[B16] Bailey KR Detecting fabrication of data in a multicenter collaborative animal study Control Clin Trials 1991 12 741 752 10.1016/0197-2456(91)90037-M 1665115

[B17] Li Q Brown JB Huang H Bickel PJ Measuring reproducibility of high-throughput experiments Annals of Applied Statistics 2011 5 1752 1779 10.1214/11-AOAS466

[B18] ENCODE Project Consortium A User’s Guide to the Encyclopedia of DNA Elements (ENCODE) PLoS Biol 2011 9 4 e1001046 10.1371/journal.pbio.1001046 21526222 PMC3079585

[B19] Langmead B Trapnell C Pop M Salzberg SL Ultrafast and memory-efficient alignment of short DNA sequences to the human genome Genome Biol 2009 10 R25 10.1186/gb-2009-10-3-r25 19261174 PMC2690996

[B20] McCullagh P Nelder JA Generalized Linear Models 1989 2 Boca Raton: Chapman & Hall/CRC

[B21] Brown BLD Zhao LH A test for the poisson distribution The Indian Journal of Statistics 2002 64 611 625

