# Supplementary material for: SERE: Single-parameter quality control and sample comparison for RNA-Seq
Source: BMC Genomics. 2012 Oct 3;13:524. doi: 10.1186/1471-2164-13-524 (PMC3534338; doi:10.1186/1471-2164-13-524)
Supplement: Additional file 1 — Contains the supplementary table and figures. [file 1471-2164-13-524-S1.pdf]

SI Table 1: SRA id and read counts for the 14 lanes of the RNA-Seq rat dataset

SI Figure 1: Reanalysis of a drosophila RNA-Seq dataset using SERE reveals variation between technical replicates

The drosophila RNA-Seq dataset (SRA id GSE17107) employed by McIntyre et al. consisted of 5 technical replicates. It was clustered using SERE and revealed 2 groups. This suggests that the 5 samples resulted from 2 libraries instead of 1 as claimed by the authors. No overdispersion is inherent within the groups. In the analysis previously conducted by McIntyre et al, Kappa was not able to pick up this inconsistency in the dataset.

SI Figure 2: Pearson's correlation coefficient varies with different total read counts.

As the total number of reads decreases, the distance between the extremes of the scale decreases similarly as indicated by the scatter-plots. Thus Pearson's *r* becomes more prone to variations and its value decreases.

SI Figure 3: Dependency of Kappa statistic on the chosen bin size

A. Kappa was 0.4109 when the singletons (exons with only one total read count) were included in the statistic. B. When the singletons were excluded from the analysis, the concordance improved and Kappa raised to 0.5087. C. When the bins were chosen tighter, Kappa decreased to 0.3018. D. Large bin sizes led to an increased concordance of 0.7272. A-D: All Kappa values represent the mean of 200 simulated RNA-Seq dataset pairs with a total read count of 10^7 (see Methods). The reads were randomly drawn into two subsets of equal size ($5x10^6$ reads in each sample) and the Kappa value for each pair was computed.

SI Figure 4: Weighted Kappa has the same limitations as Simple Kappa

A. Contamination experiment (see description Figure 1) B. Effect of the total read count (see description Figure 2) C. Effect of unequal sample sizes (see description Figure 3). A-C All values represent the mean of 200 simulated RNA-Seq dataset pairs (perfect in silico replicates).

SI Figure 5: Irreproducible discovery rate (IDR) for "duplicated" replicates. The change of correspondence curve ψ (psi) on the scale of the number of observations *t* generated by IDR shows a straight line at 1.0 for two "duplicated" replicates suggesting perfect reproducibility. Thus, the method cannot identify the underlying dataset as underdispersed ("copy and paste" replicates).

SI Figure 6: Irreproducible discovery rate (IDR) for a pair of in silico replicates for various total coverages per exon. The change of correspondence curve ψ (psi) on the scale of the number of observations *t* shows that the ranking of lowly covered exons is less reproducible (noise level) and contributes to points above 1. By increasing the required coverage per exon, these points dissapear, although they might still be within the margin of the underlying distribution type (Poisson variation).

SI Table 1

Condition	Animal	Lane	SRA ID	UMRs to genome	UMRs to exons
CONTROL	1	1	GSM539553	7741514	5512030
		2	GSM539554	7692598	5478590
		3	GSM539555	7455452	5303998
	2	1	GSM539556	8107262	5672358
		2	GSM539557	8242133	5771421
		3	GSM539558	8214866	5743284
SNL	1	1	GSM539559	6989927	4972649
		2	GSM539560	8166021	5810044
		3	GSM539561	7563536	5377969
		4	GSM539562	7002557	4968028
	2	1	GSM539563	7235937	5234429
		2	GSM539564	8310193	6009593
		3	GSM539565	8274366	5982965
		4	GSM539566	7640134	5514301

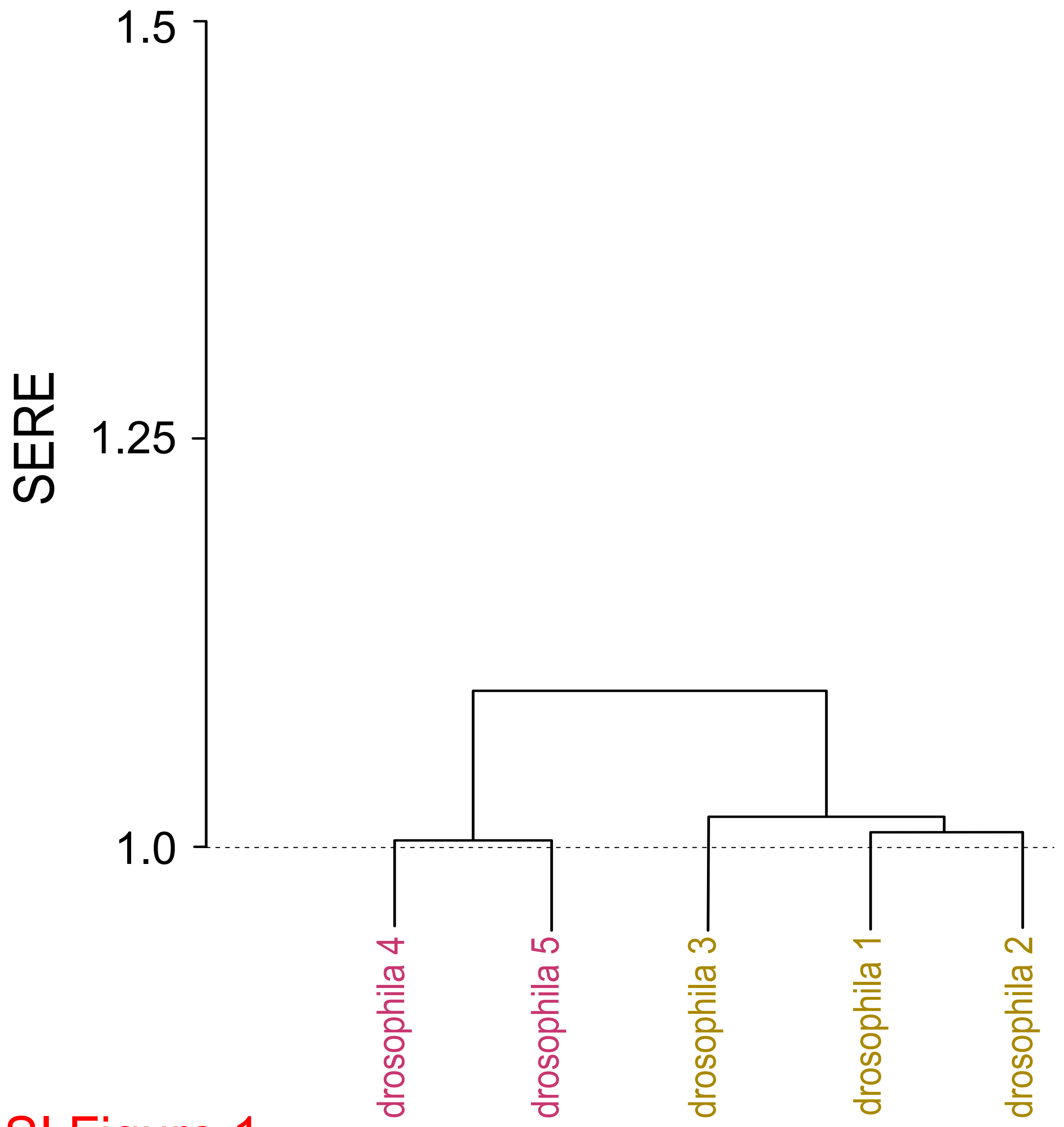

1.5
1.25
1.0
SERE
drosophila 4
drosophila 5
drosophila 3
drosophila 1
drosophila 2

SI Figure 1

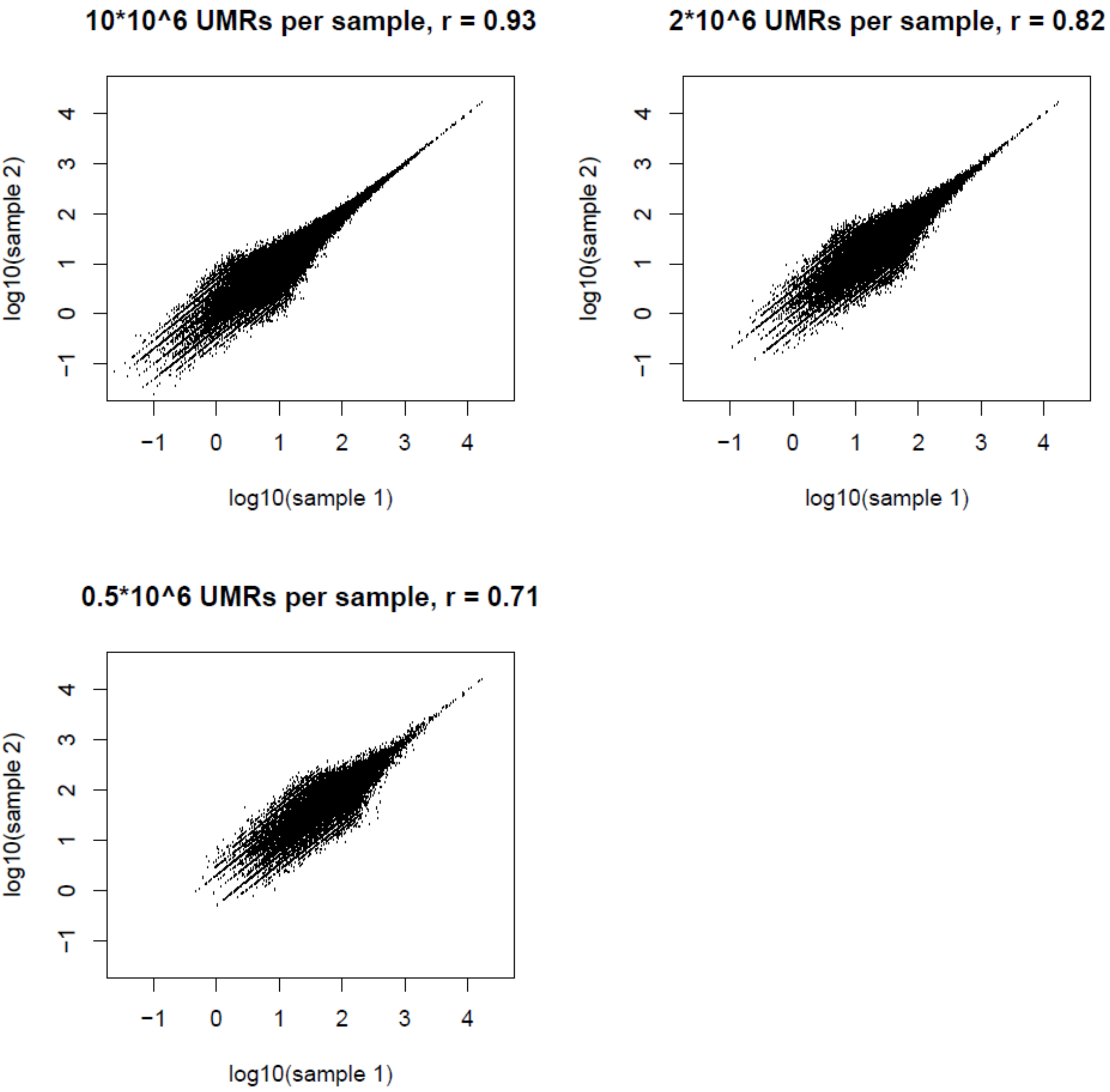
10*10^6 UMRs per sample, r = 0.93
2*10^6 UMRs per sample, r = 0.82
0.5*10^6 UMRs per sample, r = 0.71
log10(sample 2)
log10(sample 1)
−1
0
1
2
3
4

SI Figure 2

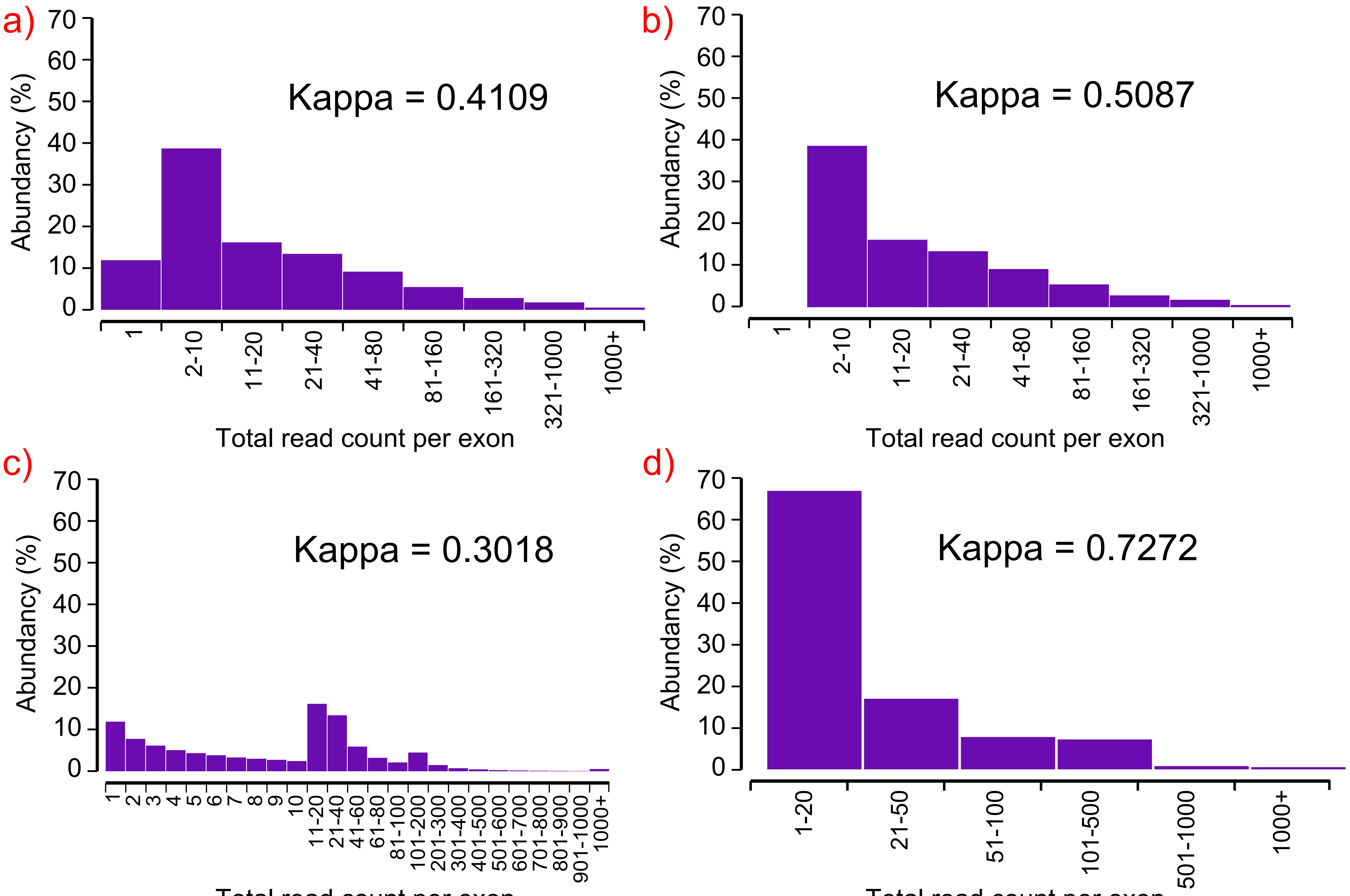
a)
Kappa = 0.4109
Abundancy (%)
0
10
20
30
40
50
60
70
1
2-10
11-20
21-40
41-80
81-160
161-320
321-1000
1000+
Total read count per exon
b)
Kappa = 0.5087
Abundancy (%)
0
10
20
30
40
50
60
70
1
2-10
11-20
21-40
41-80
81-160
161-320
321-1000
1000+
Total read count per exon
c)
Kappa = 0.3018
Abundancy (%)
0
10
20
30
40
50
60
70
1
2
3
4
5
6
7
8
9
10
11-20
21-40
41-60
61-80
81-100
101-200
201-300
301-400
401-500
501-600
601-700
701-800
801-900
901-1000
1000+
Total read count per exon
d)
Kappa = 0.7272
Abundancy (%)
0
10
20
30
40
50
60
70
1-20
21-50
51-100
101-500
501-1000
1000+
Total read count per exon

SI Figure 3

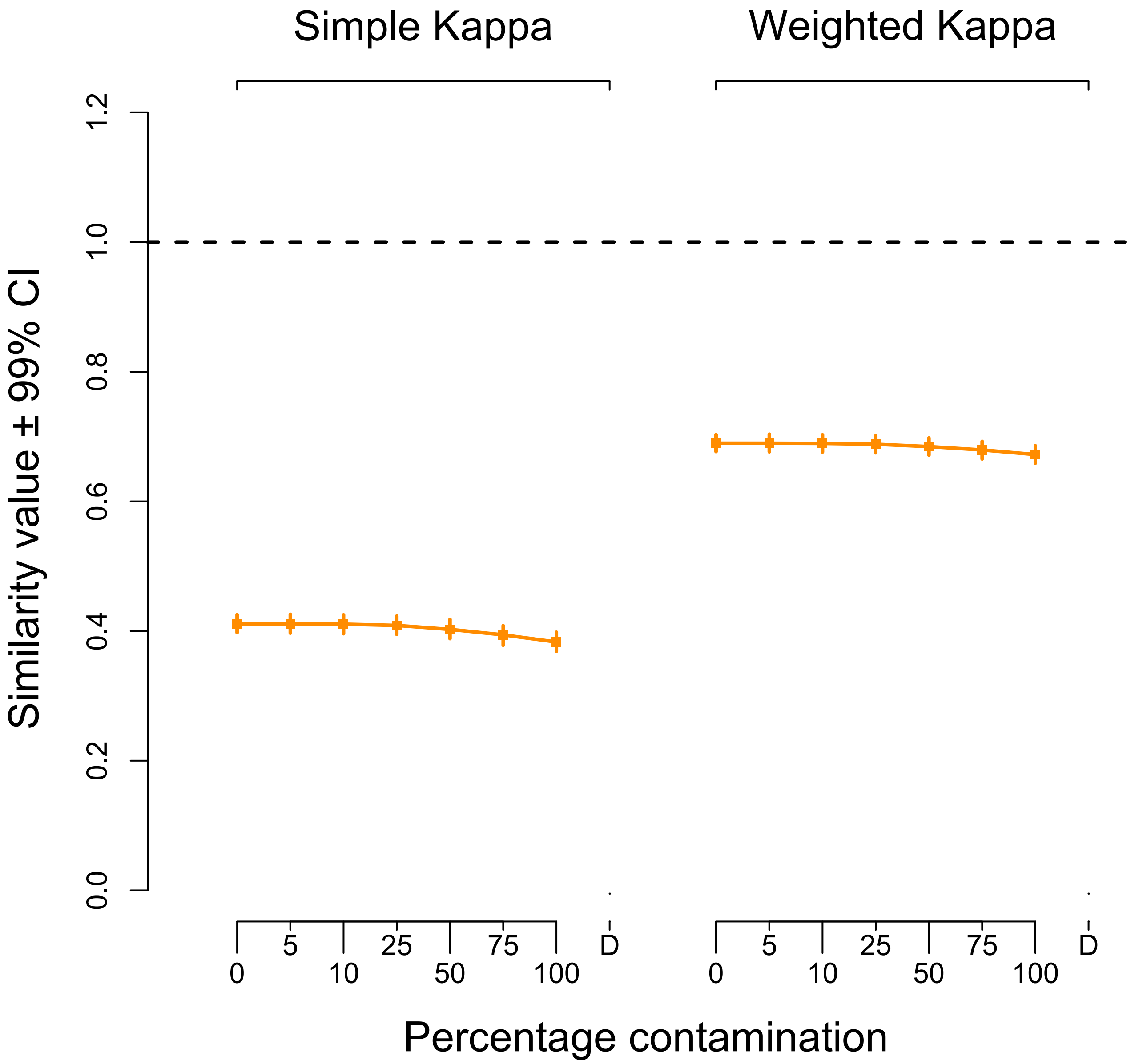
Simple Kappa
Weighted Kappa
Similarity value ± 99% CI
0.0
0.2
0.4
0.6
0.8
1.0
1.2
0
5
10
25
50
75
100
D
0
5
10
25
50
75
100
D
Percentage contamination

SI Figure 4 A

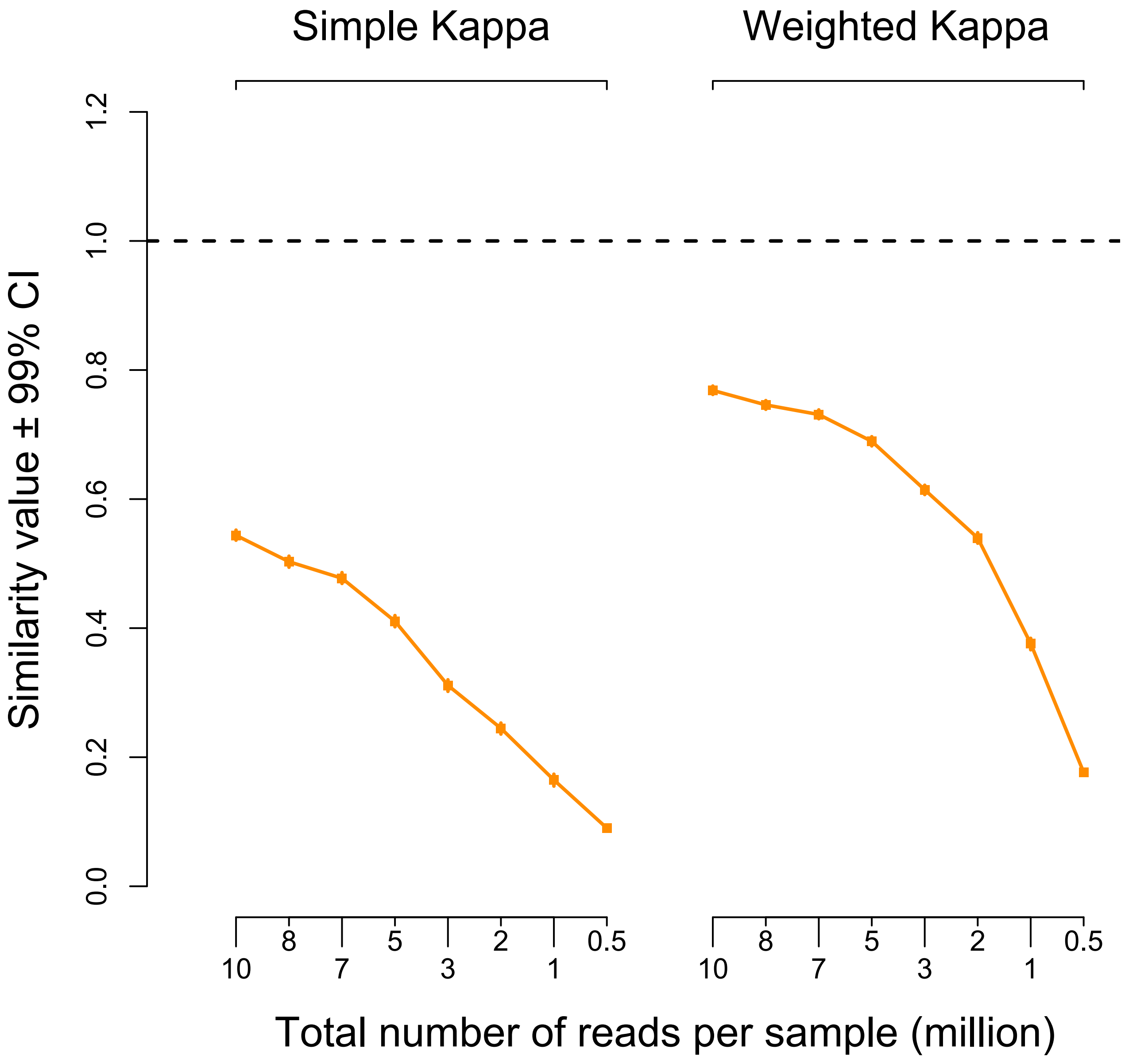

Simple Kappa
Weighted Kappa
Similarity value ± 99% CI
1.2
1.0
0.8
0.6
0.4
0.2
0.0
10
8
7
5
3
2
1
0.5
Total number of reads per sample (million)

SI Figure 4 B

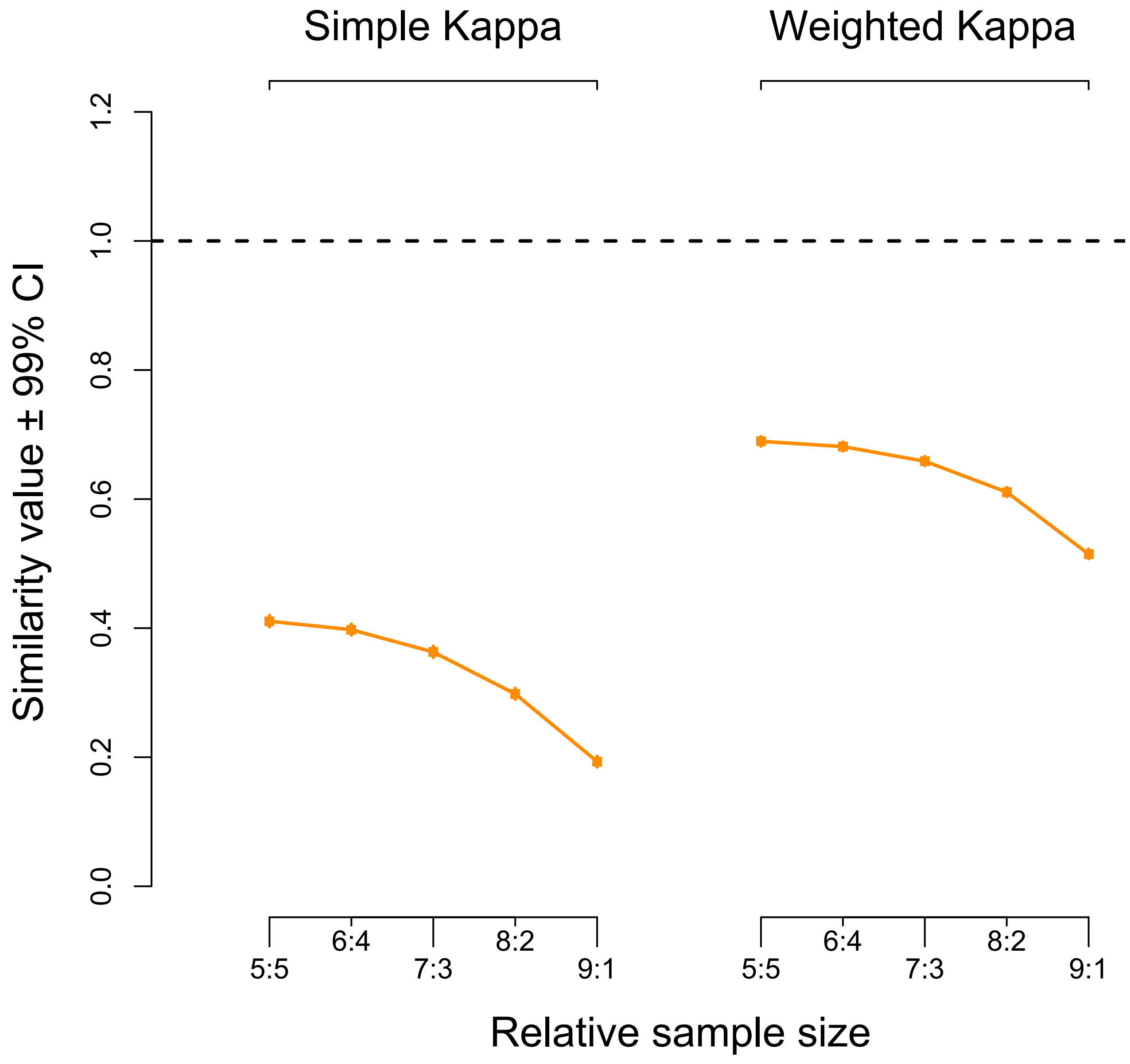

Simple Kappa
Weighted Kappa
Similarity value ± 99% CI
1.2
1.0
0.8
0.6
0.4
0.2
0.0
5:5
6:4
7:3
8:2
9:1
5:5
6:4
7:3
8:2
9:1
Relative sample size

SI Figure 4 C

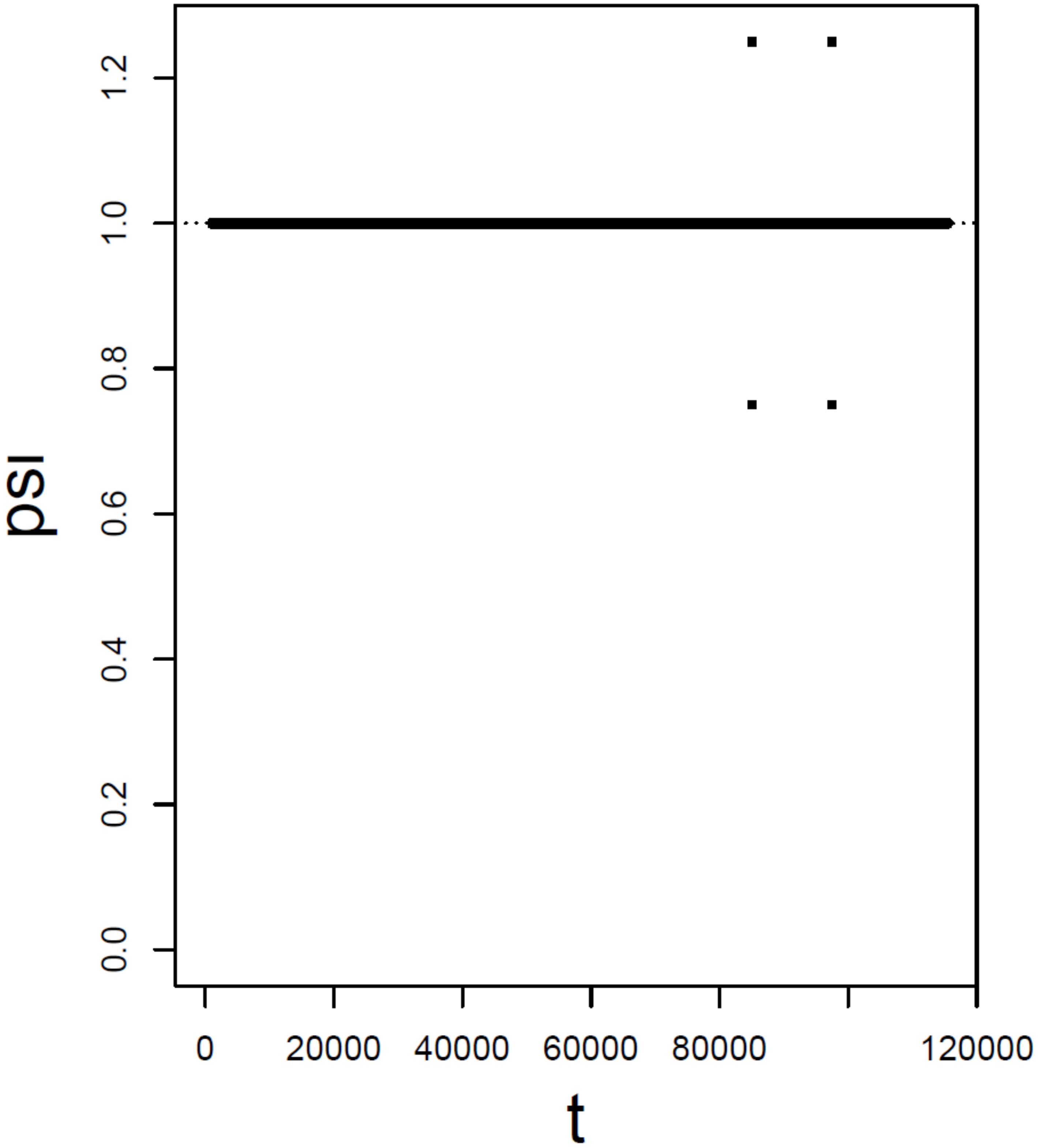

SI Figure 5

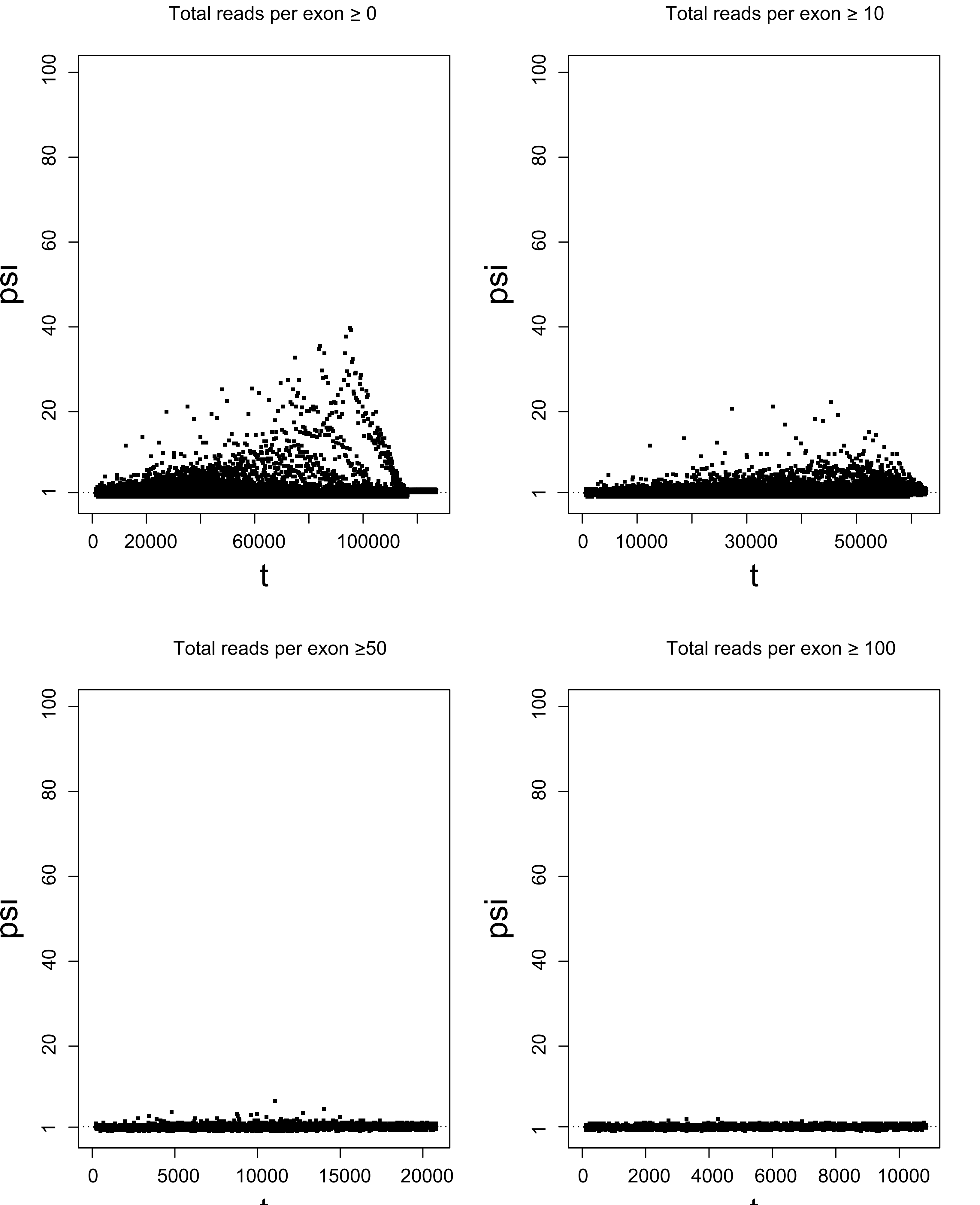
Total reads per exon ≥ 0
Total reads per exon ≥ 10
Total reads per exon ≥50
Total reads per exon ≥ 100
psi
t
100
80
60
40
20
1
0
20000
60000
100000
10000
30000
50000
5000
15000
2000
4000
6000
8000

SI Figure 6
